# The impact of membrane perforation and L-PRF for vertical ridge augmentation with a xenogeneic block graft: an experimental study in a canine model

**DOI:** 10.1007/s00784-023-05018-x

**Published:** 2023-04-21

**Authors:** Abdelrahman K. Eldabe, Khaled A. Abdel-Ghaffar, Ahmed E. Amr, Ashraf M. Abu-Seida, Ehab S. Abdelhamid, Ahmed Y. Gamal

**Affiliations:** 1 Dept. of Oral Medicine and Periodontology, Faculty of Dentistry, Assiut University, Assiut, Egypt; 2 Dept. of Oral Medicine and Periodontology, Faculty of Dentistry, Ain Shams University, Cairo, Egypt; 3 Dept. of Surgery, Anesthesiology, and Radiology, Faculty of Veterinary Medicine, Cairo University, Giza, PO: 12211 Egypt; 4 Dept. of Oral Pathology, Faculty of Dentistry, Ain Shams University, Cairo, Egypt; 5 Ain Shams University, Faculty of Dentistry, Cairo, Egypt; 6 Misr University for Science and Technology, Faculty of Dentistry, October 5 City, Egypt

**Keywords:** Growth factor, Guided bone regeneration, Perforated collagen membrane, Platelet-rich fibrin, Tissue engineering

## Abstract

**Objectives:**

This study evaluated clinically and histologically the efficacy of modified perforated collagen membrane (PCM) and/or leukocyte- and platelet-rich fibrin (L-PRF) in combination with xenogeneic block bone graft in the vertical alveolar ridge augmentation.

**Materials and methods:**

Six adult mongrel dogs were enrolled in this randomized blinded study. After defect preparation, xenogeneic screw-fixed block graft was covered by an occlusive collagen membrane in group 1 that represented the control group (Block + CM). In group 2, L-PRF membrane was added first before top coverage by occlusive collagen membrane (Block + L-PRF + CM). Groups 3 (Block + PCM) and 4 (Block + L-PRF + PCM) were identical to the first two groups except that the occlusive collagen membrane was replaced by a perforated one. Following a healing period of 2 months, the dogs were submitted to the surgical reentry phase for clinical and histological evaluation.

**Results:**

Clinically, no significant differences were found among all groups regarding vertical and horizontal ridge dimensions (*p* = 0.155, 0.492, respectively). Histomorphometric analysis revealed that the percentage of the total bone area and mature bone was significantly higher in group 4 (69.36 ± 2.72, 33.11 ± 5.18) compared to the control group (59.17 ± 4.27, 21.94 ± 2.86) (*p* = 0. 027, *p* = 0.029).

**Conclusion:**

The use of xenogenic block grafts in combination with a double-layered perforated collagen L-PRF membrane in vertical ridge augmentation appeared to improve the inductive power of this challenging defect type.

**Clinical relevance:**

Size and number of perforations may affect the mechanical and handling properties of the membrane.

## Introduction 

In vertical ridge augmentation, tissues need to grow outside the containment of bony walls which is challenging for blood clot and graft stabilization. Also, angiogenesis from pristine bone into the graft material needs to reach a certain distance which makes vertical ridge augmentation a biologically demanding procedure [1]. Clinically, graft fixation and tension-free primary wound closure require high surgeon experience due to an increase in ridge dimensions which calls for the advancement of soft tissues to provide a closed healing environment [2]. Several techniques have been used for effective vertical bone gains like using particulate bone grafts and bone graft substitutes [3]; barrier membranes with tenting for guided bone regeneration (GBR) [4, 5]; autogenous [6], xenogenic [7], and allogenic [8] block grafts; and distraction osteogenesis [9]. Despite extensive research over the last three decades, the ideal technique remains unknown, particularly in terms of the relative effectiveness of various techniques for vertical clinical bone growth (VCBG) [10]. Because of its homogeneity and regenerative ability, autogenous bone in block or particulate forms has been considered the current gold standard and the most favorable augmentation substance. However, an additional surgical site is often required for bone harvesting, which increases morbidity, healing time, and patient visits[8, 11].

Xenografts are animal-derived graft biomaterials. These inorganic bone matrix materials can be derived from bovine, porcine, and, more recently, equine sources. The crystalline structure apatite, a group of phosphate minerals, is present in all xenografts (HA, fluorapatite, and chlorapatite). The most notable advantage is that it is non-resorbable. Unlike allografts, which can lose volume over time, xenografts retain their volume [12].

The use of occlusive barrier membranes plays an important role during GBR since it prevents the down growth of the rapidly growing soft tissues and keeps a space for bone regeneration [13]. On the other hand, occlusive barrier membranes have a clear negative effect by isolating the periosteum which is considered the main source for progenitor cells [14] and osteogenic mediators [15]. Therefore, using a modified perforated collagen membrane (PCM) was suggested to allow for a positive share of periosteal progenitor cells and mediators through opening microchannels connecting the periosteum with the underlying graft materials [14, 16]. PRF (platelet-rich fibrin) as an autologous membrane was claimed to release growth factors like transforming growth factor (TGFb-1), platelet-derived growth factor (PDGF-AB), and vascular endothelial growth factor (VEGF) in addition to matrix proteins like thrombospondin-1, fibronectin, and vitronectin [17]. Leukocyte- and platelet-rich fibrin (L-PRF) was reported to slowly release biologic mediators in a sustained manner for 7 days or more [18]. L-PRF membranes have been shown to have a beneficial effect on cell proliferation in a variety of cell types, and leukocytes regulate cell responses and growth factor release [19, 20].

The main hypothesis behind this work was to utilize possible mediators of L-PRF to attract periosteal cellular and molecular components through membrane perforations in an attempt to add inductive power to xenogenic block graft in vertical ridge augmentation. The present study compared clinically and histologically the use of occlusive membranes with that of PCM with and without L-PRF combined with xenogeneic block graft in vertical ridge augmentation in a dog model.

## Material and methods

### Animal grouping and allocation

Six adult mongrel dogs (20–25 kg and 3–4 years old) with a fully erupted permanent dentition were enrolled in this experimental study. Dogs were obtained commercially from Al-Fahad Trading Company for Animals (Abu Rawash, Giza, Egypt). The animals were kept in separate kennels (1.5 m × 2.5 m × 3 m) under suitable conditions of nutrition, ventilation, a clean environment, and a 12-h light/dark cycle. All dogs were acclimatized to the housing and food for 14 days prior to the study. Dogs were fed two times per day including soft food and milk. Fresh water was supplied ad libitum.

**Fig. 1:**
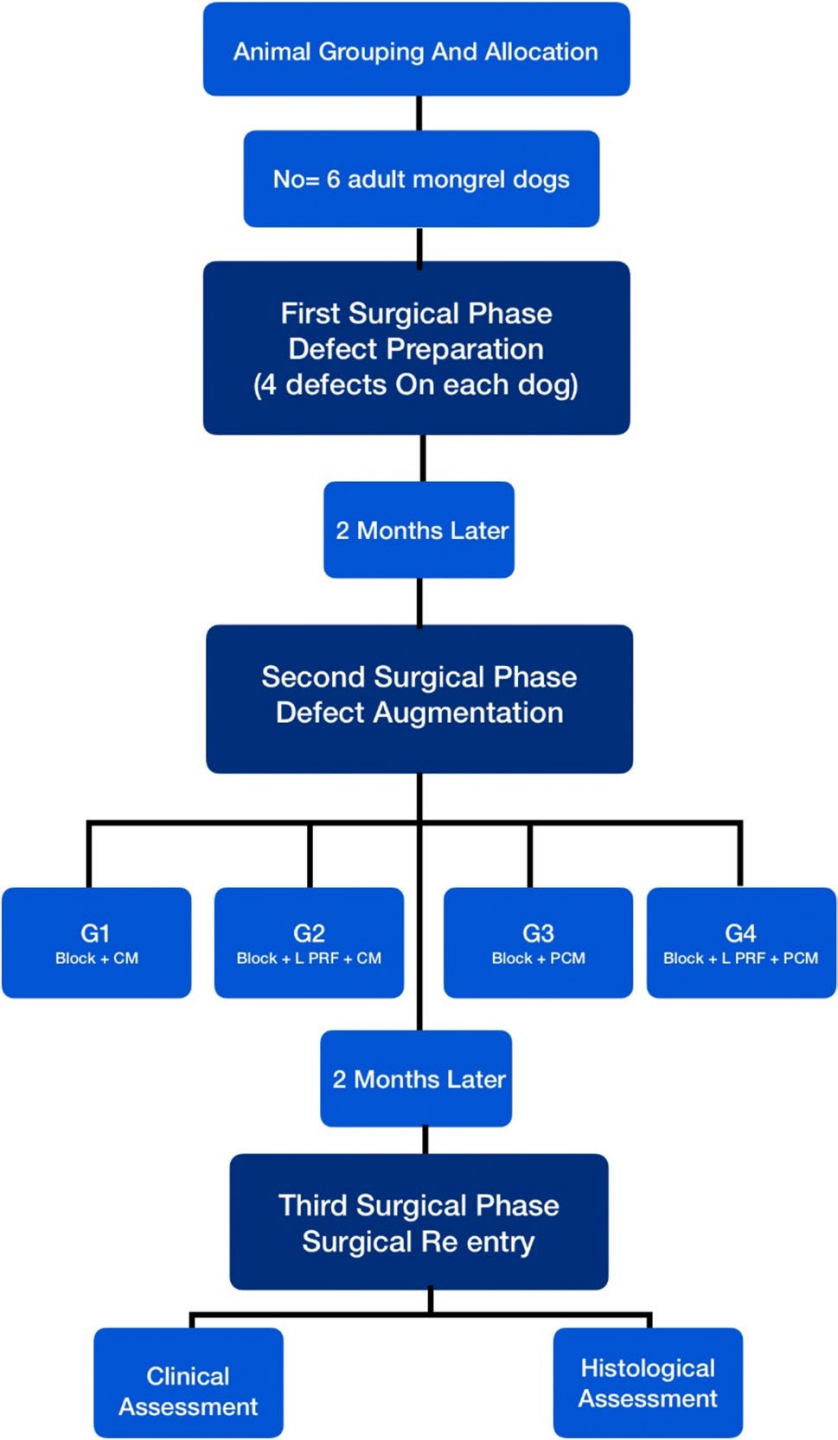
Graphic representation of the study design

This research was approved by the ethical committee at the Faculty of Dentistry, Ain Shams University, Egypt (approval number: FDASU-RecD031603). All procedures were applied according to the international regulations of animal care and use. The experiment was performed in three surgical phases: defect preparation, augmentation, and surgical reentry phases (Fig. 1). Defects were randomly allocated into four groups using balanced block randomization utilizing a computer-generated protocol. 1 In group 1, block graft was covered by a conventional occlusive collagen membrane (Block + CM), while in group 2, four compressed layers of L-PRF membrane were added first before top coverage by occlusive collagen membrane (Block + L-PRF + CM). In group 3, the block graft was covered by a perforated collagen membrane (Block + PCM), while in group 4, four compressed layers of L-PRF membrane were added first before top coverage by perforated collagen membrane (Block + L-PRF + PCM).

### First surgical phase

Dogs were pre-medicated with subcutaneous atropine sulfate^2^ at a dose of 0.05 mg/kg and intramuscular xylazine HCl^3^ at a dose of 1 mg/kg. Anesthesia was induced by intravenous ketamine HCl^4^ at a dose of 5 mg/kg and was then maintained by intravenous thiopental sodium^5^ 2.5% solution at a dose of 25 mg/kg. Routine dental infiltration anesthesia using 1.0–1.8 ml of 4% articaine hydrochloride^6^ with 1:100,000 epinephrine was used locally at the surgical site.

**Fig. 2:**
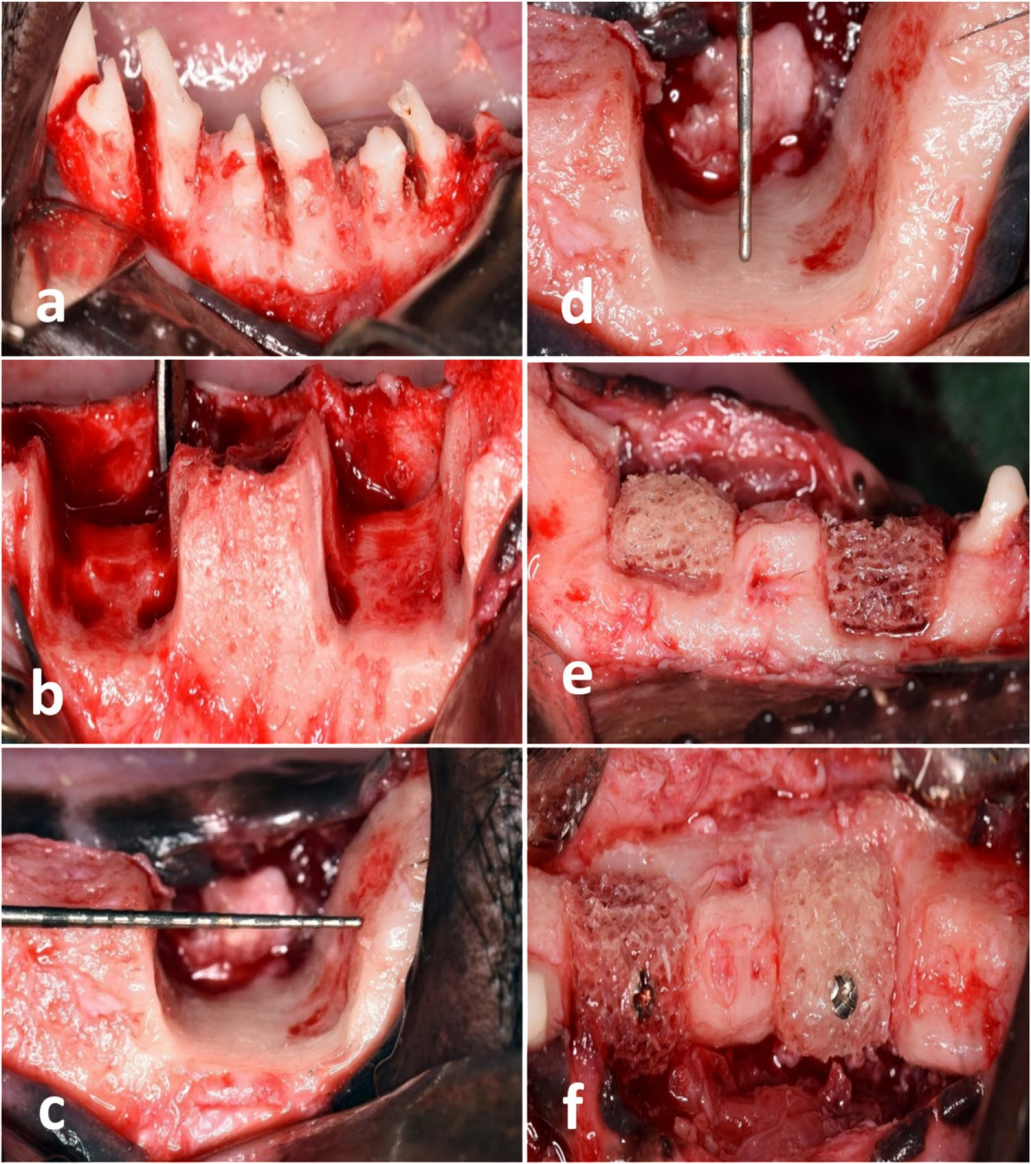
Defect creation in the first surgical phase followed by augmentation procedures in the second phase. **a** Separation of premolars before extraction. **b** Two induced mandibular vertical defects were created on each side. **c, d** All attempts were made to standardize the defects. **e, f** Bone block adjustment and fixation on the defect

In each dog, four standardized saddle-type mandibular defects (mesiodistal width: 10 mm and height: 8 mm) were planned to be prepared (two defects on each side) on the premolar area. All surgical procedures were performed by one surgeon (A. K.). A mandibular full-thickness incision was made bilaterally from the canine to the second molar. Mucoperiosteal flaps were raised at the buccal and lingual sides. The mandibular 2nd, 3rd, and 4th premolars (P2–4) were split buccolingually and extracted (Fig. 2a). After exposing the alveolar bone, four standardized saddle-type defects including the vestibular and oral aspects of the alveolar ridge were subsequently prepared using a surgical fissure carbide bur in a straight handpiece under copious saline irrigation (Fig. 2 b). Final refinement of the defect to its planned dimension was done by a back action chisel. During bone block removal, all attempts were made to standardize the size of all defects using a periodontal probe.^7^ The final dimensions of all defects were approximately 8–10 mm mesiodistally, 6–8 mm apico-coronally, and 12–14 mm buccolingually at the bottom of the defect based on the original ridge dimension. Primary wound closure was achieved by the horizontal mattress and simple interrupted sutures using resorbable (5/0) Vicryl sutures.^8^ Defects were left for 8 weeks to establish stable chronic defects and create adequate soft tissue for wound primary closure after grafting in the second surgical phase.

### Second surgical phase and L-PRF preparation

Following a healing period of 8 weeks, with the same anesthetic schedule utilized in the first surgical phase, venous blood was collected from the jugular vein in dry glass tubes and centrifuged at low speed (2700 rpm) for 12 min by using PRF-specific centrifuge.^9^ Three layers were formed: the RBC base layer, acellular plasma top layer, and L-PRF clot in the middle. By using a specific box, the L-PRF clots were pressed, and membranes were folded 4 times to be ready for their application [21]**.**

**Fig. 3:**
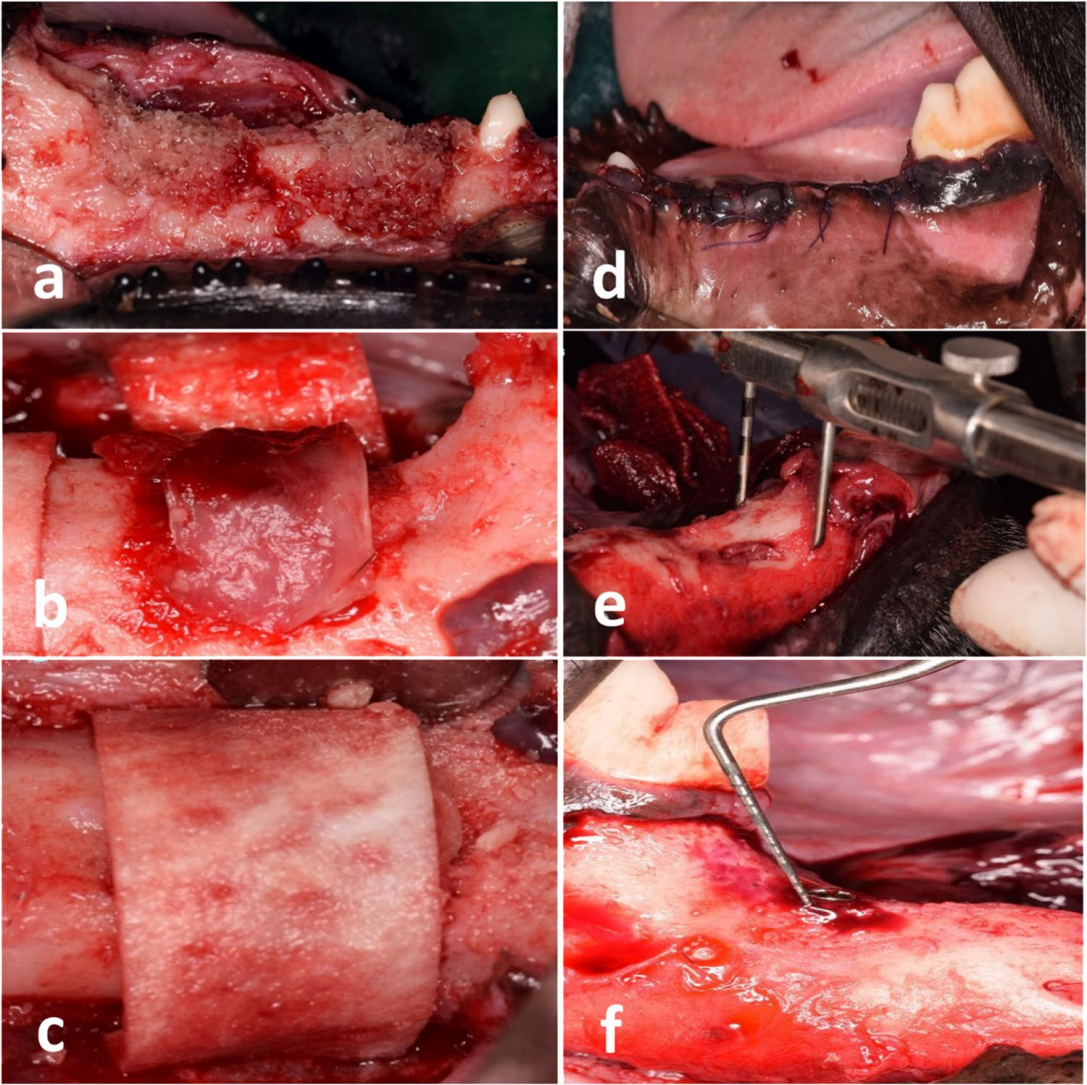
Particulate bone graft was used to fill any voids. **b** Layers of L-PRF were applied in groups 2 and 4. **c** Perforated collagen membrane utilized in groups 3 and 4. **d** Tension-free primary closure after augmentation procedures. **e** Horizontal ridge dimension (mean value of 6-point measurements of ridge width). **f** Vertical ridge dimension (exposed part of the fixation screw)

Mid-crestal full-thickness incision and periosteal reflection were performed. The four defects were subsequently exposed and readjusted to their original standardized dimensions using a surgical fissure carbide bur under copious irrigation with sterile saline (Fig. 2c, d). For each defect, a xenogeneic block graft^10^ was trimmed, adjusted, fitted, and secured in place using osteosynthesis screws 12–14 mm in length and 1.5 mm in width with a cross flat head^11^ (Fig. 2e, f). All attempts were made to adjust the screw head with the level of the neighboring bone crest. The lines of demarcation between the block and pristine bone and all voids were grafted by a xenogeneic particulate graft of the same block graft origin (Fig. 3a). The vertical dimension of the augmented defect was measured using a periodontal probe in the middle of the defect in line with the fixation screw. For group 1, after block tailoring and fixation, occlusive collagen membrane^12^ was trimmed to the appropriate shape, draped over the ridge to cover the block graft completely, and extended beyond the defect margins by approximately 3 mm. In group 2, four compressed layers of L-PRF were adapted over the block graft with 3 mm marginal extension before occlusive collagen membrane over-coverage (Fig. 3b). In group 3, collagen membrane was perforated using a 25 gauge dental before block top coverage (Fig. 3c). Interperforation spaces were determined to be ≤ 2 mm to avoid the reduction in membrane stiffness [22]. In group 4, bone block was covered first by four compressed layers of L-PRF followed by a perforated collagen membrane over-coverage.

Following each surgical procedure, all dogs were given intramuscular injections of cefotaxime sodium at a dose of 10 mg/kg^13^ and diclofenac sodium at a dose of 1.1 mg/kg^14^ once daily for five postoperative days [23].

### Third surgical phase of clinical and histomorphometric assessment

Following a healing period of 8 weeks post-grafting, animals were submitted to surgical reentry for clinical and histomorphometric assessment. Mucosal health was inspected for wound closure, oedema, purulence, or any area of exposure. Following the same anesthetic schedule used for the previous two surgical phases, a mid-crestal full-thickness incision was made in order to expose the area of interest guided by the head of the fixation screw using a back action chisel.

The primary outcomes of this study include vertical graft loss (VGL) and the amount of vertical bone gain (VBG). Secondary outcomes include percentages of new bone, marrow space, and the remaining graft particles. Also, the amount of mature bone and immature tissues was inspected.

The following measurements were assessed by an experienced calibrated examiner (A. M. A.) who was blinded about the type of intervention and not involved in any other part of the study:

Measuring the amount of vertical graft loss (VGL) was performed using a periodontal probe to measure the mean of the exposed parts of the osteosynthesis screw at their four different sides (mesial, distal buccal, lingual) (Fig. 3f). The vertical bone gain (VBG) was calculated by subtracting the exposed part of the screw from the vertical dimension of the middle part of the block graft that was measured during surgery. For horizontal augmentation assessment, a sliding bone caliper^15^ was used at sex reference points (Fig. 3e), two points at the middle (coincided with the screw), two mesial (3 mm apart from screw), and 2 distal (3 mm apart from screw). The coronal three points (3 mm from the top of the defect) were used for the assessment of the mean horizontal dimension coronally and the apical points (6 mm from the top of the defect) for the assessment of the mean horizontal dimension apically.

By the end of the 3rd surgical phase, all dogs were sacrificed by an overdose of thiopental sodium. Individual blocks containing the fixation screw and the surrounding hard tissues were fixed in 10% formaldehyde followed by decalcification via immersion in EDTA 17% solution for about 100 days. Finally, the specimens were dehydrated in a series of graded ethanol solutions. Blocks were cut in a buccolingual plane using a diamond band saw fitted into a precision slicing machine. Two histological slides were obtained from the central part of the augmented area marked by the screw (central slides). Sections were subsequently reduced to a thickness of about 50 µm using a cutting–grinding device and stained with hematoxylin and eosin (H&E) stain and Masson’s trichrome (MTC) stain.

Histomorphometric analyses and microscopic observations were performed by an experienced investigator masked to the specific experimental conditions (E. S. A.). The percentages of new bone, marrow space, and remaining graft particles were assessed. Also, the amount of mature bone and immature tissues was inspected by using Masson’s trichrome stain. For image acquisition, a color CCD camera^16^ was mounted on a binocular light microscope.^17^ Digital images with (× 40, × 100) magnifications were evaluated using an image analysis software program.^18^ Since the quantity of newly formed bone could vary along the height of the block graft, the area of interest was divided into two parts taking the fixation screw as a reference point. The upper area corresponded to the first three threads of the fixation screw (new bone—periosteal side), and the next three threads represented the apical bone area (new bone—native bone side).

For every specimen, two sections were obtained with four randomly selected fields within each section making a total of eight measurements for every specimen with their mean value representing the final value used in statistical analysis. A total of 48 slides (2 from each augmented area) were utilized for histomorphometry. A calibration procedure was initiated for the image analysis software and revealed that repeated measurements of different sections were similar at ˃95% level.

### Statistical analyses

Power analysis was performed using the G*Power software.^19^The sample size was calculated based on the primary outcome measure which was the amount of resorbed graft material. According to a previous study [24], reporting a standard deviation of 11.1, calculations based on an analysis of variance (ANOVA) test with a power = 80%, a significance level *α* = 0.05 (type I error), and effect size *f* = 0.697 yielded 5 samples for each group. By calculating an attrition rate of 20%, the final sample size was increased to be 6 animals (defects) per group. The total sample size was 24 covering the four groups and the attrition ratio.

The mean and standard deviation values were calculated for each group in each test. Data were explored for normality using the Kolmogorov–Smirnov and Shapiro–Wilk tests, and data showed parametric (normal) distribution. Clinical outcome variables included vertical graft loss (VGL), vertical bone gain (VBG), horizontal ridge dimension coronally, horizontal ridge dimension apically, and total horizontal ridge dimension (mean value of 6-point measurements of ridge width). Histological outcome variables included the percentage of new bone, marrow spaces, and remaining graft particles after image analysis. New bone—in upper (periosteal side) and apical (native bone side)—was analyzed separately. Also, the amount of mature bone and immature tissues was calculated and analyzed. One-way ANOVA followed by the Tukey post hoc test was used to compare more than two groups in non-related samples. Paired *t*-test was used to compare two groups in related samples. So it was used to compare means of horizontal ridge dimension coronally and apically in the same group. The significance level was set at *p* ≤ 0.05. Statistical analysis was performed with IBM® SPSS® Statistics version 20 for Windows.^20^

## Results

### Clinical findings

Healing proceeded uneventfully for 22 out of the 24 sites, with two sites (one in group 1 and the other in group 3) exhibiting thin overlying soft tissue revealing fixation screw head shadow during the 8 weeks following the augmentation phase (during surgical reentry). By the beginning of the augmentation phase, mandibular alveolar ridges appeared like chronic defects, simulating localized atrophic posterior mandibles. During the surgical reentry phase, all blocks appeared physically integrated with the surrounding native alveolar bone.

Table 1 shows the mean horizontal and vertical ridge dimensions at 8 weeks following augmentation for all groups. Horizontal and vertical linear ridge dimensions did not show any significant difference between all groups (*p* > 0.05). Horizontal diameter appeared significantly higher at the apical area compared to the coronal one in all groups (*p* < 0.05). Although group 4 showed the highest amount of vertical bone gain (6.83 ± 1.03), differences were not statistically significant compared to other groups (*p* = 0.155).

**Table 1:** Horizontal and vertical ridge dimensions at eight weeks following augmentation for all groups (mean ± SD)

	HRD (mm)	HRCD (mm)	HRAD (mm)	VGL (mm)	VBG (mm)
Group 1 (Block + CM) (*n* = 6)	9.11 ± 1.51	8.08 ± 1.46^a^	10.15 ± 1.69^a^	2.33 ± 1.40	5.42 ± 1.24
Group 2 (Block + L-PRF + CM) (*n* = 6)	10.37 ± 1.59	9.50 ± 1.69^b^	11.25 ± 1.76^b^	1.35 ± 0.99	6.75 ± 1.04
Group 3 (Block + PCM) (*n* = 6)	10.21 ± 1.60	9.30 ± 2.02^c^	11.16 ± 1.23^c^	1.50 ± 1.26	6.67 ± 1.37
Group 4 (Block + L-PRF + PCM) (*n* = 6)	10.21 ± 1.52	9.11 ± 1.68^d^	11.30 ± 1.40^d^	1.58 ± 1.16	6.83 ± 1.03
*p* value	0.492	0.507	0.532	0.517	0.155

*HRD* horizontal ridge dimension, *HRCD* horizontal ridge coronal dimension, *HRAD* horizontal ridge apical dimension, *VGL* vertical graft loss, *VBG* vertical bone gain

^a^Significant difference (*p* = 0.0042) between HRAD and HCAD in group 1

^b^Significant difference (*p* = 0.0259) between HRAD and HCAD in group 2

^c^Significant difference (*p* = 0.0079) between HRAD and HCAD in group 3

^d^Significant difference (*p* = 0.0004) between HRAD and HCAD in group 4

### Histological findings

**Fig. 4:**
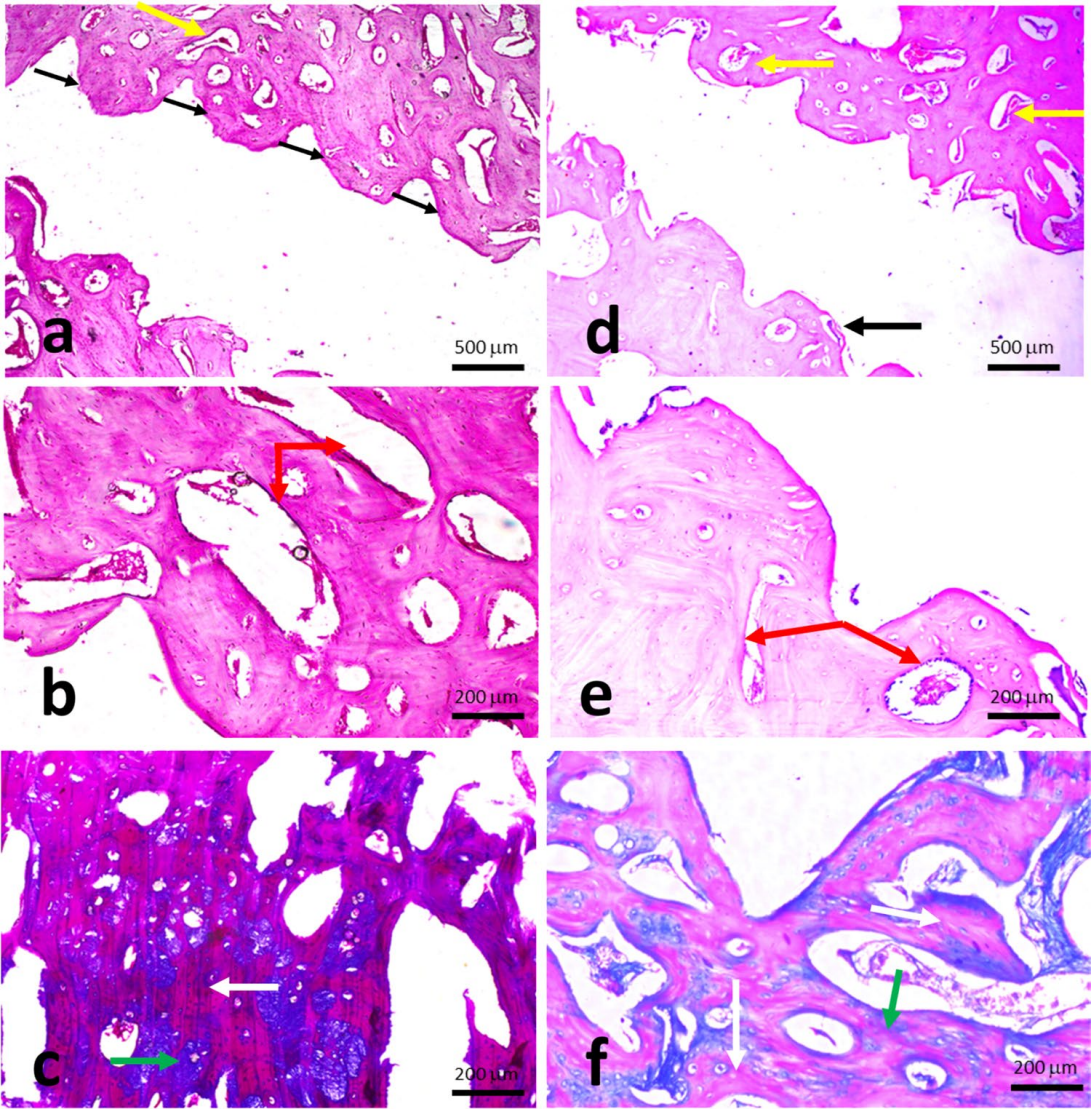
Photomicrograph of a specimen in group 1 (Block + CM) (H&E, × 4). **b** Higher magnification of the same specimen showing the new bone around the screw. (H&E, × 10). **c** Photomicrograph of the same specimen stained with Masson’s trichrome showing areas of the newly formed mature bone. Notice the red-stained mature lamellar bone developed in a patchy/isolated fashion (white arrow) on a background of the immature blue-stained bone (green arrow) (MTC, × 10). **d** Photomicrograph of a specimen in group 2 (Block + L-PRF + CM) showing the osseous tissue formed along the perimeter of the fixation screw (H&E, × 4). **e** A higher magnification of the same specimen (H&E, × 10). **f** Photomicrograph of the same specimen in group 2 stained with Masson’s trichrome showing a generalized maturation of the newly formed bone (indicated by the red MTC staining) (MTC, × 10). Black arrows refer to the fixation screw/bone interface, yellow arrow refers to marrow spaces, red arrow refers to interconnected trabeculae, white arrow refers to mature bone, and green arrow refers to immature tissues

**Fig. 5:**
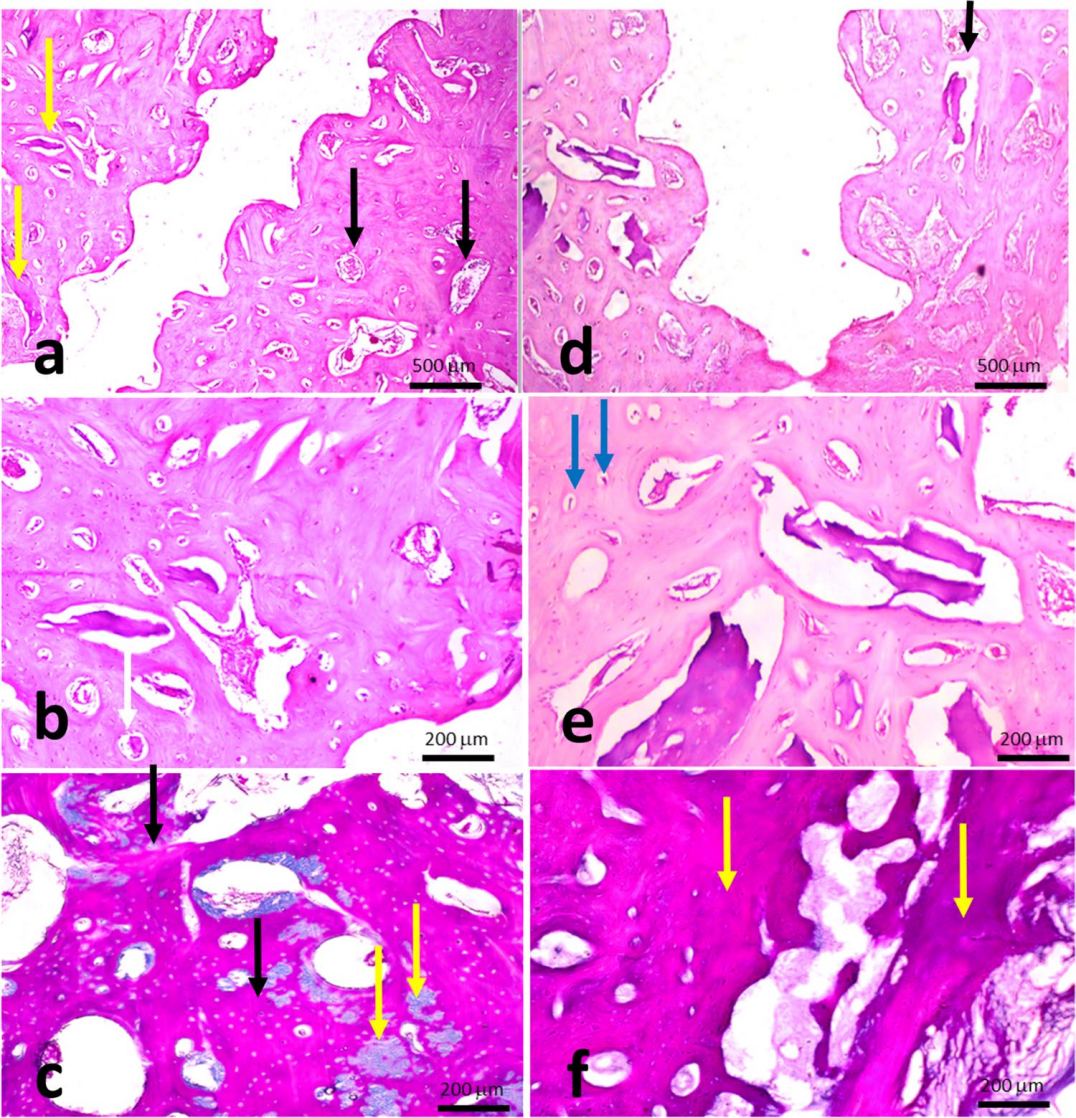
Photomicrograph of a specimen in group 3 (Block + PCM) showing dense, organized, and perfused new bone. Many of these marrow spaces show marked vascularization (black arrows). Notice the remains of grafting bone material (yellow arrows) (H&E, × 4). **b** Higher magnification of the same specimen. Notice a primary attempt toward organization into mature compact bone, through the formation of osteon complexes (white arrow) (H&E, × 10). **c** Photomicrograph of the same specimen stained with Masson’s trichrome showing an extensive maturation of the heavy newly formed bone (black arrows). The intervening blue-stained patches represent the leftover immature bone that has not consolidated yet (yellow arrows) (MTC, × 10). **d** Photomicrograph of a specimen in group 4 (Block + L-PRF + PCM) showing strikingly thick new bone trabeculae (black arrow) (H&E, × 4). **e** A higher magnification of the same specimen. Many canals display the distribution of the surrounding osteocytes and bone lamellae in an osteonal arrangement, as a sign of maturation (blue arrows) (H&E, × 10). **f** Photomicrograph of the same specimen stained with Masson’s trichrome showing a massive organization of newly formed bone into a highly mature MTC red-stained osseous tissue (yellow arrows) (MTC, × 10)

Histologically, all specimens demonstrated various degrees of bone formation coronal to the native basal bone. Different amounts of remaining graft particles were noticed along with different levels of marrow elements. Complete graft survival up to the head of the fixation screw was evident in five out of 24 specimens (1 defect in each group except group 3 with 2 defects). The new bone in groups 3 and 4 (H&E specimens) appeared dense, well organized, and perfused with bone marrow spaces of varying sizes and shapes. Many of these marrow spaces expressed marked vascularization. Using higher magnification, the new bone manifested all signs of viability, in terms of cellularity and vascularity, with osteocytes located within their lacunae and a marrow space embracing a dilated blood vessel and lined by osteoblasts (Figs. 4 and 5).

Most of the examined samples in group 4 (L-PRF and perforated membrane) stained with H&E showed new bone trabeculae strikingly thick, coherently connecting, nearly normal architecture, and infiltrated with anastomosing medullary cavities containing a highly cellular/vascular loose connective tissue stroma. Small-sized marrow spaces and vascular channels were also evident. Some basophilic remains of bone grafts were embedded within marrow cavities. There was a heavy deposition of dense collagenous tissue, with a superimposed hyalinization, to further organize into osteoid. The new bone was rich in both osteocytes and osteoblasts, which form a prominent layer lining the marrow spaces. Many canals displayed the distribution of the surrounding osteocytes and bone lamellae in an osteonal arrangement, as a sign of maturation. Higher magnification revealed the circumferential arrangement of osteocytes and the fine lamellae running in a concentric manner around the marrow cavities, suggesting the formation of osteonal complexes, as a cardinal sign of bone maturation. The same group stained with MTC mostly showed a massive organization of the newly formed bone, whether heavy compact trabeculae or short thinner ones, into a highly mature MTC red-stained osseous tissue. The immature blue-stained bone was very sparse and hardly detectable. The dense collagen bundles around the newly formed bone took up the MTC blue staining. Minimal spaces for adipose tissue were observed in some marrow spaces in both groups.

Table 2 shows the histological characteristics for all groups at 8 weeks following surgery. For total bone percentage, a statistically significant higher level was found in group 4 compared to group 1 (*p* = 0.027). No significant difference was reported between group 2 and group 3 with that of the control. Group 4 (Block + L-PRF + PCM) showed statistically significant higher percentages of total bone at the periosteal level (71.20 ± 2.75) compared to the control group (58.32 ± 3.46) where *p* = 0.023. Percentage of bone marrow spaces (BMS) and remaining graft particles (RGP) in all groups revealed no statistically significant differences (*p* = 0.786 and 0.087, respectively). Masson’s trichrome bone maturity stain revealed a statistically significant higher percentage for group 4 compared to group 1 (*p* = 0.029). No significant differences were found between other groups and that of the control. Regarding immature tissues, there was a significantly higher level in group 1 (34.34 ± 2.54) compared to group 3 (22.83 ± 4.72) and group 4 (23.54 ± 5.15) (*p* = 0.002 and 0.004, respectively).

**Table 2:** Histological outcomes (mean ± SD)

	TB (%)	BMS (%)	RGP (%)	BP (%)	BA (%)	MB (%)	IT (%)
Group 1 (Block + CM) (*n* = 6)	59.17 ± 4.27^a^	17.67 ± 4.26	7.36 ± 2.66	58.32 ± 3.46^b^	63.19 ± 4.34	21.94 ± 2.86^c^	34.34 ± 2.54^d,e^
Group 2 (Block + L-PRF + CM) (*n* = 6)	62.41 ± 8.71	18.51 ± 4.47	6.37 ± 2.20	65.58 ± 11.44	64.81 ± 9.48	24.82 ± 6.10	28.32 ± 5.67
Group 3 (Block + PCM) (*n* = 6)	64.53 ± 5.34	17.63 ± 6.18	9.84 ± 2.59	65.82 ± 6.86	69.98 ± 7.29	29.43 ± 9.36	22.83 ± 4.72^d^
Group 4 (Block + L-PRF + PCM) (*n* = 6)	69.36 ± 2.72^a^	15.78 ± 3.67	8.15 ± 1.38	71.20 ± 2.75^b^	74.19 ± 4.86	33.11 ± 5.18^c^	23.54 ± 5.15^e^
*p* value	0.039*	0.786	0.087	0.038*	0.050	0.030*	0.001*

*TB* total bone percentage, *BMS* bone marrow space, *RGP* remaining graft particles, *BP* bone periosteal, *BA* bone apical, *MB* mature bone, *IT* immature tissue

^*^Significant difference between the four groups (*p* < 0.05)

^a^Significant difference (*p* = 0.027) between group 1 and group 4 at TB (%)

^b^Significant difference (*p* = 0.023) between group 1 and group 4 at BP (%)

^c^Significant difference (*p* = 0.029) between group 1 and group 4 at MB (%)

^d^Significant difference (*p* = 0.002) between group 1 and group 3 at IT (%)

^e^Significant difference (*p* = 0.004) between group 1 and group 4 at IT (%)

## Discussion

The study’s main objective was to investigate the possibility of adding more inductive power for xenogenic block graft in bone augmentation using L-PRF as a possible source of biologic mediators which could aid in attracting periosteal osteogenic cells and mediators through membrane perforations. Given the body of scientific evidence from the eligible studies included in a recent systematic review regarding the effectiveness of vertical ridge augmentation interventions, no clear conclusions can be drawn regarding the superiority of any particular VRA technique [13, 25–27]. Although an autologous bone graft is regarded as the gold standard, it is associated with limited availability, morbidity at the donor site, and unpredictable resorption [28, 29]. Onlay xenogenic block grafting is one of the most reliable techniques [30] that could help in solving the problems associated with autogenous grafts. Its physicochemical structure was found similar to that of natural bone and showed osteoconductive properties [31]. The usage of block graft guarantees more wound stability and space maintenance capacity when compared with the particulate form of graft.

To the best of our knowledge, no previous studies compared the double membrane coverage of perforated collagen membranes and L-PRF to conventional occlusive ones in xenogenic block ridge augmentation therapies. The surgical defect dimensions used in this study are well accepted and documented in many studies as a non-spontaneous healing critical-sized defect for the evaluation of bone grafts, either alone or in combination with membrane placement and growth factors used [32–34].

The clinical results clearly showed that all the intervention techniques were successful for vertical ridge augmentation with uneventful healing in nearly all sites. Surgical reentry revealed that linear bone measurements were almost close in all groups with a non-significant difference in both horizontal and vertical bone dimensions. This expected outcome could be attributed to the non-resorbable nature of the used xenogenic block grafts. The resorbability of the xenogenic bone mineral is questionable, and usually, it is very slow and takes a very long time if it happens [30, 35].

Group 4 gains the highest although insignificant vertical bone gain value compared to the other treated groups (6.83 ± 1.03). Possible mediators of L-PRF could help in periosteal elements’ chemoattraction through membrane perforations. In a recent retrospective study by Amaral Valladão et al., leukocyte- and platelet-rich fibrin was used in conjunction with a mixture of particulate autogenous and xenogenic grafts in a proportion of 1:1 for staged vertical and horizontal ridge augmentation. The horizontal bone gain was an average of 5.9 ± 2.4 mm, while the vertical one was 5.6 ± 2.6. The authors suggested that PRF added to the bone graft could improve the angiogenesis, the migration of stem cells, and the osteogenic differentiation in the whole graft, favoring the graft’s integration and clinical results [36]. In one study that used perforated collagen membrane and particulate allograft in horizontal ridge augmentation, up to 5 mm of clinical and radiographic horizontal bone gain was reported [37]. The authors compared their outcomes with that of a systematic review and meta-analysis that reported a mean horizontal bone gain of 3.9 mm. The greatest bone gain width was reported to be 5.7 mm using autogenous bone combined with particulate xenograft and a resorbable membrane. Up to 3.5 mm of lateral bone gain width was reported in this systematic review using a particulate allograft and resorbable membrane which demonstrated the added value of perforated membrane usage [38]. Simion et al. highlighted the importance of the periosteum as a source of progenitor cells during bone regenerative procedures. They used xenogenic equine block graft with no membrane coverage in combination with recombinant human platelet-derived growth factor-BB (rhPDGF-BB) for vertical ridge augmentation in critical size defect of dogs. Vertical bone regeneration was clearly predictable and consistent when rhPDGF-BB was added to each block matrix without the imposition of a barrier membrane. The authors concluded that growth factor-mediated bone regeneration benefited when access to the periosteum was not prohibited by a barrier membrane [39].

Histologically, a statistically significant higher total bone percentage and amount of mature bone were reported in group 4 compared to group 1. The amount of newly formed bone was significantly higher in the periosteal bone side of the defect in group 4 compared with the control group (*p* = 0.023). Membrane macropores were reported to allow for the migration of periosteal cells, mediators, and stem cells to the defect area to positively share in the healing process [40]. These histological outcomes correspond with a study by Gutta et al. who applied cortico-cancellous tibial bone to the lateral border of the mandible protected with barrier membranes. The experiment analyzed three different pore-sized membranes and a control occlusive membrane. Macroporous membranes showed a significantly higher amount of bone formation than any other groups [41]. Recently, a study by Aristodemou et al. showed the role of the perforated membrane in enhancing the regenerative capacity at critical size defect created in diabetic rats. Non-significant difference was observed between the uncontrolled diabetic group (47.8%) and the healthy group (63.6%) regarding mineralized tissue formation. When occlusive membrane was utilized, significant osseous formation was found in the healthy group. The authors justify this result by the role of perforations in allowing the migration of osteogenic cell populations from the neighboring supra-calvarial tissues (including the periosteum) and from the dura mater into the wound. The presence of such osteogenic cells might have masked the potentially negative effect of the underlying uncontrolled diabetes, which, on the other hand, was evident when occlusive barriers were employed [42].

Gamal et al. studied the optimal pore diameter that allows for maximum attraction of periosteal progenitor cells and mediators. A pore diameter of 0.7 mm is reported to show the highest mesenchymal stem cell migration and proliferation [43]. Lorenz et al. claimed that the addition of the L-PRF-based matrices to bone graft might result in accelerated migration of osteoprogenitor cells in the augmentation bed through bioactive growth factor release, thus increasing the regenerative capacity of the bone graft itself [44].

It is important to highlight that the present study has some limitations like the high regenerative power in dogs compared to that of humans, the limited sample size, the single follow-up, and the lack of standardization of membrane perforations.

Within the limitations of the present study, we can conclude that perforated collagen-augmented L-PRF membranes revealed more favorable results regarding total bone and amount of mature bone when compared to the control group (Block + CM). Growth factor-mediated bone regeneration gained by L-PRF and periosteal cellular and molecular components could be compromised by the use of occlusive barrier membranes. Clinical evaluation of this double membrane effect and xenogenic block grafts in vertical ridge augmentation needs to be evaluated.

## Footnotes

1 Excel; Microsoft, Redmond, USA.

2 Atropine sulfate 1%®, ADWIA, Egypt.

3 Xylaject 2%®, ADWIA, Egypt.

4 Keiran®, EIMC Pharmaceuticals Co., Egypt.

5 Thiopental sodium®, EIPICO, Egypt.

6 Artinibsa 40 mg/ml, Inibsa Dental, Spain.

7 CP15; Hu-Friedy Co., Chicago, IL, USA.

8 Assucryl,Assut sutures, Switzerland.

9 IntraSpin System, Intra-Lock, USA.

10 Bio-Gen Block, Bioteck, Arcugnano (Vicenza)—Italy.

11 Aesculap, Inc. Hazelwood, USA.

12 Hypro-sorb, Bioimplon GmbH, Friedrich, Germany.

13 Cefotax 250 mg vial®, T3A Co., Egypt.

14 Voltaren 75 amp®, Novartis Co., Egypt.

15 MEDESY srl, Industrial Area, Italy.

16 Color View III, Olympus, Hamburg, Germany.

17 Olympus BX50, Olympus, Germany.

18 ImageJ 1.49b, NIH, USA.

19 G*Power, Ver. 3.192 copyright 1992–2014, Düsseldorf, Germany.

20 IBM SPSS Statistics for Windows, version 20.0. Armonk, NY: IBM Corp, USA.
